# Enhancing the stress responses of probiotics for a lifestyle from gut to product and back again

**DOI:** 10.1186/1475-2859-10-S1-S19

**Published:** 2011-08-30

**Authors:** Susan Mills, Catherine Stanton, Gerald F Fitzgerald, R Paul Ross

**Affiliations:** 1 Teagasc Food Research Centre, Moorepark, Fermoy, Co. Cork, Ireland; 2 Department of Microbiology, University College Cork, Ireland; 3 Alimentary Pharmabiotic Centre, Cork, Ireland

## Abstract

Before a probiotic bacterium can even begin to fulfill its biological role, it must survive a battery of environmental stresses imposed during food processing and passage through the gastrointestinal tract (GIT). Food processing stresses include extremes in temperature, as well as osmotic, oxidative and food matrix stresses. Passage through the GIT is a hazardous journey for any bacteria with deleterious lows in pH encountered in the stomach to the detergent-like properties of bile in the duodenum. However, bacteria are equipped with an array of defense mechanisms to counteract intracellular damage or to enhance the robustness of the cell to withstand lethal external environments. Understanding these mechanisms in probiotic bacteria and indeed other bacterial groups has resulted in the development of a molecular toolbox to augment the technological and gastrointestinal performance of probiotics. This has been greatly aided by studies which examine the global cellular responses to stress highlighting distinct regulatory networks and which also identify novel mechanisms used by cells to cope with hazardous environments. This review highlights the latest studies which have exploited the bacterial stress response with a view to producing next-generation probiotic cultures and highlights the significance of studies which view the global bacterial stress response from an integrative systems biology perspective.

## Introduction

To suggest that probiotic bacteria have entered the realm of the highly exploited bacterial groups is an understatement. Moreover, man’s expectations of probiotic bacteria have perhaps become the most demanding for any bacterial group to date. With numerous health-promoting attributes, many of which have been scientifically validated in animal and human clinical trials, probiotic bacteria have become an integral element to everyday healthy living. Indeed, the global market for probiotics is estimated to exceed US$28.8 billion by 2015 [1]. Probiotic bacteria generally execute their biological role in the gut, which can involve a plethora of effects from immunomodulation to production of bioactive metabolites, all of which have dramatic consequences for disease evasion [2-4]. The present target of any probiotic food product in terms of probiotic cell numbers is to have up to 10^7^ colony forming units (cfu)/g at the end of its shelf life [5], although it should be emphasized that this will probably become strain and application specific as new clinical evidence emerges supporting health claims. Thus, before probiotic bacteria can even begin to fulfill their physiological role in the gut, the bacteria themselves must endure a number of stresses to ensure they reach the target site in adequate numbers to elicit an effect. These stresses can be considered in two distinct contexts; firstly, probiotic bacteria must be processed in suitable form to enable oral consumption and secondly the bacteria must be able to withstand the harsh conditions imposed during passage through the gastrointestinal tract (GIT).

## From gut…

Potential probiotic cultures have been isolated from a variety of sources including animal, human and food sources. However, there is now growing evidence that strains are host specific [6] and for that reason it is generally recommended that human isolates should be used for human applications as a starting point. In this respect human strains have been isolated from various sites from oral to stool samples.

## To product…

In terms of processing, probiotics are commonly grown to high numbers before undergoing a drying process to produce a high-cell density probiotic powder. This can then be added to a specific carrier, such as a dairy product, which is stored under suitable conditions. Probiotic robustness can be compromised even in the initial growing stages. Indeed, Muller et al. [7] recently demonstrated that the nutrient composition of the growth medium can have a significant effect on the technological properties of probiotics by altering membrane composition and morphology. The presence of linoleic and linolenic acids in a minimal growth medium reduced the acid and heat tolerance of *Lactobacillus johnsonii* NCC533 by 6- and 20-fold, respectively [7]. The stresses encountered during drying include extremes in temperatures, from very high temperatures in spray-drying, to very low-temperatures during freeze drying and storage, as well as osmotic and oxidative stresses. During spray-drying temperatures can reach as high as 200^o^C [8] and while the exposure time for the cells is extremely short, the integrity of viable bacterial cells can be severely compromised. The cytoplasmic membrane is the primary target for heat damage, resulting in damage to fatty acids and aggregation of proteins, however, intracellular proteins, ribosomes and RNA can also be duly impaired [9,10]. In addition to heat stress, spray-drying also exposes cells to osmotic stress, dehydration and oxidative stress [10]. At the other end of the scale, cells are typically frozen at -196^o^C during freeze-drying, and then dried by sublimation under high vacuum [11-13]. Freeze-drying is less harsh on cells than spray-drying resulting in higher cell viability [14]. However, the low temperature still compromises cellular integrity where the main consequences include reduction in membrane fluidity, increases in the rate of DNA strand breakage, stabilization of RNA and DNA secondary structures which in turn alters transcription, translation and replication [15]. Ribosome functions, protein folding and enzyme activity are also disturbed at low temperatures [16,17]. Oxygen stress can affect probiotic bacteria at various stages of their production, from fermentation to drying and even storage etc. While oxygen itself does not damage the cells, its partial reduction to water generates reactive oxygen species (the superoxide anion radical O_2_^-^, the hydroxyl radical OH^•^, and hydrogen peroxide H_2_O_2_) which can lethally damage proteins, lipids and DNA [5,18]. Cysteine in the active sites of enzymes can also be a target for oxidation [19]. Oxygen sensitivity can be particularly problematic for bifidobacteria, although variations have been observed across this species in terms of oxygen tolerance [20,21]. Indeed, two species isolated from porcine caecum, *Bifidobacterium aerophilum* and *Bifidobacterium psychroaerophilum*, were found to be aerotolerant with the ability to grow on agar-medium under aerobic conditions [22]. Certain strategies have proven successful in terms of reducing the effects of oxygen toxicity. For example, electro-reduction, de-aeration or addition of reducing agents to non-fermented pasteurized milk was shown to enhance the viability of bifidobacteria during extended storage [23]. In fermented milk, co-cultivation of *Bifidobacterium longum* BB536 with *Lactococcus lactis* ssp. *lactis* MCC866, improved the survival of the bifidobacterial strain [24]. In this case, *L. lactis* ssp. *lactis* MCC866 was found to express several genes involved in protection against active oxygen species at higher levels during refrigerated storage, compared to non-effective lactococcal strains. Moreover, the food matrix itself can impose multiple stresses on bacterial survival. For example, several genes contributing to stress-related functions were induced in the commercial meat starter *Lactobacillus sakei* 23K during meat fermentation [25]. The global transcriptional response of *Lactobacillus reuteri* ATCC 55730 to the sourdough environment also revealed the up-regulation of various stress-related genes during sourdough fermentation [26]. Probiotic viability in Cheddar cheese was found to be markedly reduced when cells were added just before cheddaring but cell losses were halved when the probiotic was added to milk before renneting [27]. Moreover, in the same study salt was found to negatively compromise cell viability. However, food matrix stresses may also contribute to the overall survival of probiotic bacteria. Leverrier et al. [28] demonstrated that freeze-dried propionibacteria included at the beginning of milk fermentation were much more robust when exposed to acid and bile salt stresses as opposed to reconstituted freeze-dried cells which were dramatically injured under the same stress conditions.

## And back again…

Passage of probiotics through the mammalian GIT is a hazardous journey, with the initial stages designed to jeopardize the survival of pathogenic microorganisms. The principal stresses include shifting pH encountered in the stomach resulting from gastric acid, as well as bile, a digestive secretion of the hepatic system which serves to emulsify and solubilize lipids and lipid soluble vitamins for metabolism, and steep gradients of oxygen. Exposure to acid negatively affects the proton motive force (PMF) across the membrane as a result of the accumulation of protons inside the cell [29]. In addition to cell membrane structural damage, acid stress causes damage to DNA and proteins [15]. Exposure to bile disrupts the integrity of the cell membrane, affects DNA, RNA structure formation as well as protein folding and exposes the cell to oxidative stress and low intracellular pH [30,31].

## The probiotic stress response

**Figure 1:**
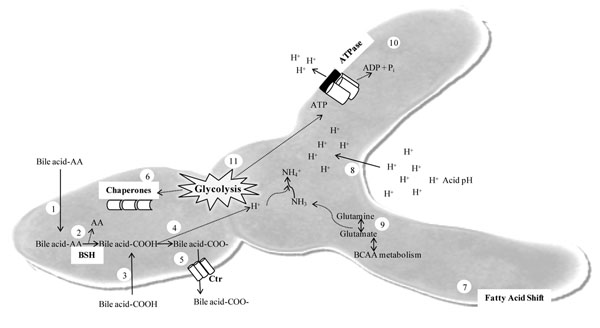
Diagrammatic representation of the main stress responses to acid pH and bile salt in bifidobacteria. (1) Conjugated bile acids enter the bacterial cytoplasm and are cleaved by BSH (bile salt hydolase) (2) releasing one amino acid and one de-conjugated bile acid moiety. (3) De-conjugated bile acid can also enter the cell by passive diffusion and becomes deprotonated (4). (5) Ionized bile salts are non-permeable and are excreted by the action of certain transporters e.g. Ctr (cholate transporter) of *Bifidobacterium longum*. (6) Synthesis of molecular chaperones is also increased and a shift in fatty acid composition of cell membrane can occur (7). Exposure to acid pH or bile salt deprotonation results in acidification of the cytoplasm (8). This can be counteracted by the production of ammonia from glutamine deamination (9) or pumping of protons from the cell by the F_1_F_0_-ATPase (10). ATP required for driving these systems is generated through glycolysis (11) (reproduced from Sanchez et al. [138]).

Bacterial cells are naturally equipped with a plethora of defense mechanisms to enhance survival in hostile environments [15,29,32-34]. These include chaperone proteins which assist the folding of misfolded proteins, proteases which degrade proteins which are irreversibly damaged, transport systems to maintain correct osmolarity, catalases and superoxide dismutases to tackle reactive oxygen species, as well as proton pumps, decarboxylases and transporters to combat decreases in intracellular pH [29] (Table 1, Figure 1). Understanding the intricacies of these systems provides scientists with a plethora of molecular tools to improve bacterial endurance. For example, a recent patent was filed describing numerous stress-related proteins of the probiotic strain *Lactobacillus acidophilus* and genetic engineering approaches which may be used to improve the stress response of the strain [35].

**Table 1:** Examples of Proteins and Genes Involved in the Stress Responses of Probiotics

Stress	Protein/Gene/System (General Role/Description)	Fold Induction	Strain	Ref
Heat	GroEL (chaperone protein)	+49.1	*Lactobacillus paracasei* NFBC338	[37]
Heat Osmotic	GroEL (chaperone protein)	+15 +3	*Lactobacillus rhamnosus* HN001	[118]
Heat	*clpL1* (Clp ATPase family, members act as chaperones and regulators of proteolysis)	+20	*Lactobacillus rhamnosus* E800	[119]
Heat, Cold, Ethanol	*hsp18.5* (heat shock protein) *hsp19.3* (heat shock protein)	nd	*Lactobacillus plantarum* Lp90	[41]
Heat, Cold, Ethanol	*hsp18.55* (heat shock protein)	nd	*Lactobacillus plantarum* Lp90	[42]
Heat	DnaK (chaperone protein) GroEL (chaperone protein) ClpE (Clp ATPase family, members act as chaperones and regulators of proteolysis)	+4.4 +3.8 +1.7	*Lactobacillus gasseri* ATCC 33323	[120]
Heat	FtsH (protease and chaperone activity)	+8	*Lactobacillus plantarum* WCFS1	[121]
Heat	HtrA (protease and chaperone activity) GroEL (chaperone protein) DnaK (chaperone protein) GrpE (chaperone protein)	+10 -+15 +5 -+10 +10 -+15 +2 -+5	*Bifidobacterium longum* 3A	[122]
Heat	*groEl-cspA*, (chaperone protein-cold shock protein) *groES* (chaperone protein)	+12 +8	*Bifidobacterium breve* UCC 2003	[123]
Heat	*clpC* (Clp ATPase family, members act as chaperones and regulators of proteolysis)	+15	*Bifidobacterium breve* UCC 2003	[124]
Heat	*clpP* operon: *clpP1*, *clpP2* (Clp ATPase family, members act as chaperones and regulators of proteolysis)	+15	*Bifidobacterium breve* UCC 2003	[125]
Heat Osmotic	*hsp20* (heat shock protein)	+28 +25	*Bifidobacterium breve* UCC 2003	[126]
Temperature downshift	*cspA* (cold shock protein)	+20	*Lactobacillus bulgaricus* VI104	[127]
Cold	ClpP (Clp ATPase family, members act as chaperones and regulators of proteolysis) Pyruvate kinase Glycoprotein endopeptidase	≥2	*Lactobacillus acidophilus* RD758	[128]
Temperature downshift	*cspL* (cold shock protein)	+17	*Lactobacillus plantarum* NC8	[46]
Osmotic	*dnaK* operon: *dnaK*, *grpE*, *dnaJ* (chaperones), ORF1 (similarity to predicted heat-controlled transcriptional regulators of Mer family)	+15, +14	*Bifidobacterium breve* UCC 2003	[129]
Osmotic	*dnaJ_2_* (chaperone)	+4.5	*Bifidobacterium breve* UCC 2003	[130]
Oxygen	NADH oxidase & NADH peroxidase		*Lactobacillus acidophilus* 2400 *Lactobacillus acidophilus* 2409 *Bifidobacterium lactis* 1941 *Bifidobacterium pseudolongum* 1944 *Bifidobacterium longum* 1944	[103]
Oxygen	AhpC (alkyl hydroperoxide reductase C22) PNDR (Pyridine nucleotide-disulfide reductase) Dps (DNA-binding ferritin-like protein) NrdA (ribonucleotide reductase) MutT1 (NTP phosphohydrolases)	+10 +2 +1.7 +2 +1.8	*Bifidobacterium longum* BBMN68	[104]
Bile	HtrA (protease and chaperone activity) DnaK (chaperone protein) GroEL (chaperone protein)	+1.5-+2 +1.5-+2 +1.5-+2	*Bifidobacterium longum* 3A	[122]
Bile	Bsh1 (bile salt hydrolase)		*Lactobacillus plantarum* WCFS1	[131]
Bile	Ctr (cholate transporter)		*Bifidobacterium longum* NCIMB 702259T	[132]
Acid	*clpL* (Clp ATPase family, members act as chaperones and regulators of proteolysis) Ir1516 (putative cell wall-altering esterase)	+2-+3 >5	*Lactobacillus reuteri* ATCC 55730	[133]
Acid	GrpE (chaperone protein)	>3.5	*Lactobacillus sanfranciscensis* CB1	[134]
Acid	*LBAI524HK* (histidine kinase)		*Lactobacillus acidophilus* NCFM	[135]
Acid	*F_0_F_1_-*ATPase operon (involved in ATP synthesis and proton extrusion)	+2	*Lactobacillus acidophilus* NCFM/N2	[136]
Acid	*F_0_F_1_-*ATPase operon (involved in ATP synthesis and proton extrusion)	+15	*Bifidobacterium lactis* DSM10140	[137]

The aim of this review is to highlight the latest studies which take advantage of the bacterial stress response to produce ‘super-fit’ bacteria through genetic manipulation. Moreover, the upsurge in genome sequencing alongside systems biology approaches is now enabling scientists to view the global cellular response to the stresses encountered during processing and transit in the gut. As well as providing a deeper understanding of the probiotic stress response, this information is generating a wealth of novel molecular tools, which may find themselves as central players in the science of probiotic enhancement in the future.

## Taking advantage of the bacterial stress response - genetic manipulation

### Exploiting probiotic stress responses

**Figure 2:**
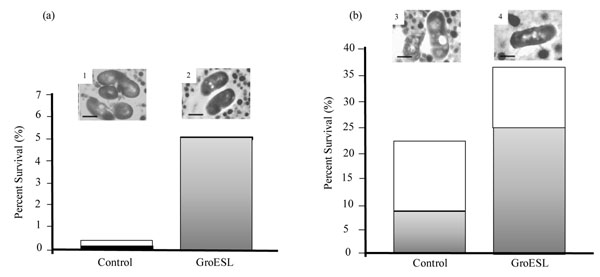
Survival of *Lb. paracasei* NFBC 338 (control) and GroESL-overproducing *Lb. paracasei* NFBC 338 (GroESL) following spray-drying (a) and freeze-drying (b). White bars represent powders plated on MRS and shaded bars represent powders plated on MRS containing NaCl (5% wt/vol). Insets show transmission electron micrographs of *Lb. paracasei* NFBC 338 (1) and GroESL-overproducing *Lb. paracasei* NFBC 338 (2) following spray-drying, and *Lb. paracasei* NFBC 338 (3) and GroESL-overproducing *Lb. paracasei* NFBC 338 (4) following freeze-drying. GroESL-overproducing *Lb. paracasei* NFBC 338 appeared to be more robust following both drying procedures compared to the control strain (adapted from Corcoran et al. [5]).

It is well accepted that adaptation to a sub-lethal dose of a specific physical or chemical stress can dramatically improve subsequent performance in compromising environments and as a consequence is a popular strategy to increase both the technological and gastrointestinal robustness of a strain. The success of this strategy is owed to the induction of the bacterial stress response during the adaptation process whereby a specific system may be induced with the result that the cells survive a previously lethal dose of the same stress, or a more general system may be targeted enabling cross-protection against a range of stresses. For example, in order to improve the viability of the probiotic strain *Lactobacillus paracasei* NFBC 338 during spray-drying Desmond et al. [36] demonstrated that pre-stressing the culture by exposure to 52^o^C for 15 minutes improved survival of the strain 700-fold (in reconstituted skim milk) during heat stress and 18 fold during spray-drying compared to un-adapted cells. Exposure to salt stress also afforded a level of thermotolerance. Indeed, following exposure to 0.3 M NaCl survival of the strain improved by 16-fold during spray-drying [36]. A proteomics analysis of the biological response of *Lb. paracasei* NFBC 338 to heat adaptation revealed an altered level of expression of at least 12 proteins, where expression of the chaperone GroEL was most dramatically induced in the test culture [37]. GroEL works in concert with its co-chaperone GroES where the complex ensures the correct folding of proteins in an ATP-regulated manner under normal growth conditions and under conditions of stress [29]. The genes encoding GroESL are negatively regulated by HrcA, which binds to a pallindromic operator sequence called CIRCE (controlling inverted repeat of chaperone expression). Subsequently, overexpression of the *groESL* operon of *Lb. paracasei* NFBC 338 using the nisin controlled expression system in *Lb. paracasei* resulted in increased survival during exposure to stressful levels of heat, salt and butanol [37]. Moreover, *Lb. paracasei* over-expressing GroESL exhibited 10-fold better survival during spray-drying and 2-fold better survival during freeze-drying demonstrating that the GroESL chaperone is an effective molecular target for enhancing the technological performance of probiotic lactobacilli during spray-drying [5] (Figure 2). Interestingly, mRNA transcripts of the chaperones GroEL and DnaK were recently up-regulated 2-fold by down-regulating the negative regulator HrcA using anti-sense RNA technology [Kearney et al. unpublished]. However, although the anti-sense strain exhibited an increased exponential growth rate compared to the control, the anti-sense strain remained as sensitive as the control strain to heat, acid, bile solvent and osmotic stresses.

The ATP-independent chaperones, the small heat shock proteins (sHsps), have also been associated with enhanced bacterial survival during stress. While these proteins are not involved in protein re-folding they are necessary for normal cellular functions including growth, stability of DNA and RNA and they prevent the formation of inclusion bodies [38-40]. Heat, cold and ethanol stresses were previously shown to enhance the expression of the heat shock genes *hsp18.5*, *hsp19.3* and *hsp18.55* encoded on the genome of *Lactobacillus plantarum*[41,42]. Interestingly, *hsp18.5* and *hsp19.3* genes were preceded by an inverted repeat at the 5’ end with homology to the operator sequence CIRCE [41] whereas the 5’ region preceding *hsp18.55* revealed the presence of putative *cis* elements able to bind alternative sigma factor σ^B^[42]. Inactivation of *hsp18.55* revealed that the protein encoded by the gene may be involved in the recovery of stressed cells during the early stage of high temperature stress [43]. Indeed, the Δ*hsp18.55* mutant strain displayed a longer lag phase under conditions of short intense heat stress (50^o^C). Physical parameters were also disturbed in the mutant strain including membrane fluidity and surface properties. Overproduction of each of the three heat shock proteins in *Lb. plantarum* improved the growth of the genetically-modified cultures during heat shock (37^o^C or 40^o^C) and cold shock (12^o^C) and overproduction of Hsp18.55 and Hsp19.3 also improved survival during solvent stress (1% v/v butanol or 12% v/v ethanol) [44].

Cold shock proteins (Csps) are induced as a result of cold shock pre-treatment where they have been associated with the stabilisation of mRNA [45]. Three induced Csps were previously identified in *Lb. plantarum* following cold shock treatment, namely CspL, CspP, CspC [46]. Overproducing each of the three proteins in *Lb. plantarum* resulted in improved performance during treatments involving a down-shift in temperature and under conditions of nutritional deprivation [47]. For example, overproduction of CspC led to faster growth when stationary phase cells were added to fresh medium when compared to controls, suggesting a role for CspC in nutrient adaptation. Cells overproducing CspL did not suffer a reduction in growth rate following exposure of exponentially growing cells to cold shock (8^o^C), while overproduction of CspP resulted in enhanced survival during 6 cycles of freeze-thawing at -80^o^C resulting in a 4-fold survival level.

### Exploiting stress responses of pathogenic microorganisms

Patho-biotechnology is a relatively new concept which aims to exploit stress response systems of pathogenic bacteria for biotechnological and biomedical purposes [48]. Indeed, pathogenic bacteria can be a rich reservoir of sophisticated mechanisms which ensure bacterial endurance in some of the most stressful environments including the host immune system or those encountered during food processing. For example, certain bacterial species accumulate protective compounds called compatible solutes as part of the stress response [49]. These compounds are generally highly soluble with no net charge at physiological pH [50]. Within the cells they serve as osmotic balancers [51] but also serve to protect enzyme function against the stresses of high temperature, salinity, freeze-thawing and drying [52,53]. The trimethylammonium compound glycine betaine is used as a compatible solute by the majority of bacteria including *Listeria monocytogenes*[54]. In this respect, the secondary glycine betaine transporter of *L. monocytogenes*, BetL, has been shown to improve survival of the strain in certain foods [55], as well as improve its osmotolerance, cryotolerance and barotolerance [56-58]. Interestingly, heterologous expression of BetL from *L. monocytogenes* in the probiotic strain *Lactobacillus salivarius* UCC118 using the nisin controlled expression system resulted in a genetically modified strain with improved resistance to numerous stresses [54]. The BetL^+^ strain accumulated 65 times more betaine than the BetL^-^ strain and was capable of growth in 7% NaCl, unlike the control which was unable to grow efficiently at the same concentration. Repeated freeze-thaw cycles at -20^o^C or -70^o^C demonstrated that the BetL^+^ strain was two logs greater than the control at -20^o^C and 0.5 logs greater at -70^o^C. The barotolerance of the test strain also improved yielding a 2-log increase over the control strain following exposure to 350 MPa. Moreover, the BetL^+^ strain survived freeze-drying and spray-drying much more efficiently than the control with a 36% survival level for test versus 18% for control following freeze-drying and a 1.4% survival level for test versus 0.3% for control after spray-drying. Cloning the *L. monocytogenes* BetL system into the probiotic strain *Bifidobacterium breve* UCC2003 significantly improved the tolerance of the strain to gastric juice and elevated osmolarity [59]. In addition, BetL^+^*B. breve* was recovered at much higher levels from the faeces, intestines and caecum of BetL^+^ inoculated mice compared to control mice inoculated with the BetL^-^ strain, and *Listeria*-challenge experiments revealed that BetL^+^ mice exhibited reduced levels of *Listeria* infection in the spleen compared to controls [59].

The marked ability of *L. monocytogenes* to survive the stresses encountered in the upper small intestine has been linked to its capacity to endure high bile concentrations. Indeed, *L. monocytogenes* has been isolated from the human gallbladder [60,61]. A novel bile resistance mechanism, termed BilE was identified in *L. monocytogenes* and operates by excluding bile from the cell [62]. Heterologous expression of BilE in *L. lactis* NZ9800 and *B. breve* UCC2003 resulted in modified strains with enhanced capacities to survive in the presence of bile [63]. For example, the *bilE*-containing strain of *L. lactis* exhibited a 2.5 log enhanced resistance (compared to control) following a 20 minute exposure to 1% porcine bile, a similar result was observed for the *bilE*-containing strain of *B. breve*. In addition, both genetically modified strains exhibited an enhanced ability to survive *in vivo* conditions using mouse models. This was particularly noteworthy for the *bilE*-containing *L. lactis* strain considering that it is generally not considered to be a gut inhabitant due to its poor survival rates in the GIT. Indeed, the BilE^+^*L. lactis* strain was detected at high levels in murine faeces 3 days after inoculation whereas the BilE^-^*L. lactis* strain was undetectable in the faecal samples on day 2 after inoculation. While both BilE^+^ and BilE^-^ strains of *B. breve* colonised the murine GI tract at relatively similar rates (10^7^ cfu/g of faeces), the persistence of the BilE^+^ strain began to differ significantly 12 days after inoculation. Indeed, by day 19 the BilE^-^ strain had reached a level of 1 x 10^5^ cfu/g of faeces, whereas the BilE^+^ strain persisted at a level of 4.5 x 10^7^ cfu/g of faeces. Moreover, by day 19 the levels of the BilE^+^ strain in the small intestine, caecum and large intestine were significantly higher compared to the control strain. This was a particularly important finding for the small intestine, a region of the GIT which has the highest levels of bile [64]. Indeed, the persistence levels of the genetically modified strain were 100 fold greater than the persistence levels of the control. The BilE^+^ strain also enhanced clearance of *L. monocytogenes* from the liver at significant levels when compared to the control strain.

### Exploiting generic microbial mechanisms

**Figure 3:**
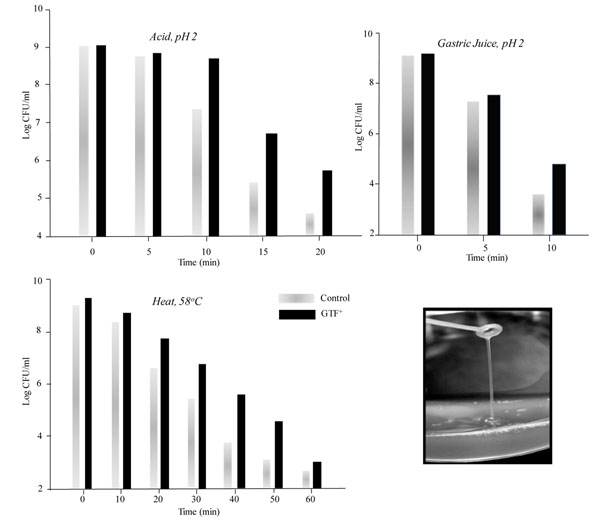
Survival of *Lb. paracasei* NFBC 338 expressing the *gtf* gene (GTF^+^) and control strain in the presence of acid, gastric juice and elevated temperature. Inset displays the loop-touch test of *Lb. paracasei* NFBC 338 producing EPS demonstrating the ‘ropy’ phenotype of the modified strain (adapted from Stack et al. [67]).

Improving probiotic robustness can also be achieved by looking to other microbial mechanisms which may not be necessarily associated with pathogenic survival. For example, various studies have demonstrated that probiotic robustness can be significantly enhanced by adding a protectant to the growth medium prior to exposure of the strain to environmental stress. An exudate gum from trees, gum acacia, enhanced survival of *Lb. paracasei* NFBC 338 from the stresses of heat, bile and H_2_O_2_ when added to the growth medium and also enhanced survival during spray-drying [65]. We proposed that the protective effect of gum acacia may be related to cellular encapsulation by the polysaccharide thus protecting the cells from the harsh environmental conditions. Many microorganisms produce exopolysaccharides (EPS) which can be either excreted into the medium or form a capsule around the cell, the capsular polysaccharides (CPS). Recently, a positive correlation was observed between EPS production and resistance to bile salt and low pH stress in *Bifidobacterium* species isolated from breast milk and infant faeces [66]. Interestingly, both the technological and gastrointestinal durability of *Lb. paracasei* NFBC 338 was significantly improved by equipping the strain with an inherent ability to produce the EPS beta-glucan [67]. The gene encoding this particular EPS, the membrane-associated glycosyltransferase enzyme (*gtf*) comes from *Pediococcus parvulus* 2.6. Indeed, heterologous expression of the gene in *Lb. paracasei* increased its heat tolerance 60-fold, its acid-tolerance 20-fold, its ability to survive in simulated gastric juice by 15-fold and its ability to survive in bile by 5.5 fold, compared to the control strain (Figure 3). Moreover, beta-glucan has been linked with many health-promoting properties including an ability to lower serum cholesterol levels [68,69], immunomodulation [70,71], anticancer properties [72] and it behaves as a prebiotic [73]. Thus, as well as enhancing the gastrointestinal robustness of the strain Stack et al., [67] simultaneously improved its health-promoting properties.

Other microbial mechanisms have also proven worthwhile as tools for probiotic enhancement. Heterologous expression of the manganese superoxide dismutase gene (*sodA*) from *Streptococcus thermophilus* in the intestinal strains *Lactobacillus gasseri* and *Lb. acidophilus* enabled the cells to tolerate greater concentrations of H_2_O_2_ (up to 1.6 mM) compared to the control which could not adequately tolerate concentrations greater than 1.2 mM [74]. Integration of *sodA* into the chromosome of *Lb. gasseri* also dramatically improved its oxidative tolerance, whereby the modified strain was capable of surviving up to 45 mM H_2_O_2_ after 90 minutes of exposure, unlike the control strain which was unable to tolerate concentrations greater than 25 mM [75]. Moreover, cloning the catalase gene *katA* from *Lb. sakei* SR911 into catalase-deficient *Lb. planatarum* TISTR850 under a strong lactococcal promoter improved the oxidative tolerance of the modified strain markedly [76]. Indeed, the modified strain survived 6 logs better than the control following 60 hours of growth under oxidative stress. Likewise, cloning *katA* from *Lb. sakei* into *Lactobacillus rhamnosus* AS 1.2466 significantly improved the oxidative resistance of the recombinant strain [77]. The exponential-phase culture of the modified strain increased 600-fold following H_2_O_2_ challenge and the stationary-phase culture increased 1000-fold under the same conditions when compared to the control. Endowing the probiotic strain *Lactobacillus casei* with both *katA* (from *Lb. sakei*) and the bile salt hydrolase gene *bsh1* (from *Lb. plantarum*) dramatically enhanced both the oxidative tolerance and the bile salt resistance of the strain [78]. The survival ratio of the recombinant strain was 40-fold greater than the control after 8 hours of incubation in the presence of 8 mM H_2_O_2_, while the recombinant strain survived in the presence of 0.5% glycodeoxycholate reaching 10^5^ cfu/ml, a concentration which killed the control cells.

## New players involved in the probiotic stress response

Proteomics and whole genome DNA microarrays alongside heterologous expression studies and the generation of deletion mutants continue to provide important insights into the response and adaptation of probiotic bacteria to environmental stresses. Indeed, studies which employ such techniques continually highlight the importance of the main molecular defense mechanisms, reveal stress-associated regulatory networks, but also provide insight into novel systems which serve to protect the stressed cell providing new molecular tools for probiotic enhancement. A global analysis of the transcriptomes of two heat-shock tolerant strains of *B. longum* (isolated following serial passages at heat-shock temperatures) demonstrated that overexpression of the *dnaK* operon and *clpB* protease-encoding gene was linked to point mutations in the gene encoding the negative regulator, HspR [79]. Indeed, in one mutant a tyrosine residue was replaced by a histidine in the helix-turn-helix domain of the regulator, while in the second mutant a tyrosine residue was replaced with a cysteine in the winged 1 motif of the protein. Sensitivity to heat was restored by complementing the mutant strains with the wild-type *hspR* gene. Interestingly, the arginine deiminase (ADI) pathway of *Lactobacillus fermentum* IMDO 130101, a strain isolated from sourdough, was shown to respond to salt and temperature stresses resulting in an increase in ornithine production [80]. The ADI pathway functions to produce extra ATP and aid with acid stress [80]. The authors suggest that the main function of the ADI pathway at high temperature is to provide energy to stationary phase cells and improve energy generation for growing cells under osmotic stress.

Heterologous expression of myosin-cross reactive antigen (MCRA), from *B. breve* NCIMB 702258, in *Lactococcus* and *Corynebacterium* improved sensitivity of both recombinant strains to heat and solvent stresses suggesting that this FAD-dependent fatty acid hydratase is involved in protecting the cell from environmental stress [81]. In addition, deletion of MCRA in *Lb. acidophilus* NCFM resulted in reduced growth of the deletion mutant in the presence of acids, bile and salt and significantly reduced the ability of the mutant strain to adhere to Caco-2 cells suggesting a role for the gene in the stress response, cell division and Caco-2 cell adherence [82]. Poly P granules are polyanionic inorganic biopolymers of orthophosphate residues which have been associated with stress responses in bacteria [83]. The putative polyphosphate kinase gene of *Bifidobacterium scardovii* responsible for poly P synthesis was recently linked to the oxidative stress response as well as providing protection against other environmental stresses such as low pH [83].

Highest survival of *Lb. casei* ATCC 334 to acid stress was achieved by exposing the cells to pH 4.5 for 10 or 20 minutes [84]. Whole genome DNA microarrays revealed the up-regulation of 104 genes and the down-regulation of 216 genes after 20 minutes at pH 4.5. Malolactic fermentation and histidine accumulation were also revealed as important features of acid adaptation in *Lb. casei*. Malolactic enzyme was up-regulated 16-fold and 7 fold following 5 and 20 minutes of acid exposure, respectively. This enzyme functions to decarboxylate L-malate to L-lactate and CO_2_, thus contributing to alkalinization of the cytoplasm and enabling the production of ATP through H^+^-ATPase [85,86]. The transcriptome of cells exposed to acid for 20 minutes also revealed the up-regulation of an eight gene cluster involved in histidine biosynthesis. The authors postulated that histidine accumulation may contribute to the buffering capacity within the cell and is the first report that histidine accumulation may enhance acid resistance in bacteria. Interestingly, addition of either 30 mM histidine or 30 mM malate during acid exposure (pH 2.5) for 60 minutes or 2 hours improved cell survival more than 100-fold or more than 10^7^-fold, respectively. The activity of the LuxS-mediated quorum sensing system, which is responsible for generating the universal signaling molecule called autoinducer-2 (AI-2) was found to be significantly increased following exposure to acid shock in *Lb. acidophilus* NCFM and *Lb. rhamnosus* GG suggesting a role for the *luxS* gene in the acid stress response of lactobacilli [87]. The gene encoding the universal stress protein Usp1 was also recently suggested to be an important mediator in the acid stress response of *Lb. plantarum *[*88*]. Indeed, studies in *Escherichia coli* demonstrated that Usp1 from *Lb. plantarum* inactivates the negative regulator PadR which is involved in the phenolic acid stress response by negatively regulating *padA*, a gene which encodes the phenolic acid decarboxylase enzyme.

A whole genome DNA microarray was employed to determine the transcriptional response of *Lb. reuteri* ATCC 55730 to bile [89]. Differential expression of a wide variety of genes involved in cell envelope stress, protein denaturation and DNA damage was observed. Interestingly, survival of *Lb. reuteri* in the presence of bile was decreased by mutating a Clp chaperone, a putative esterase and a gene of unknown function, whereas the ability of the strain to restart growth in the presence of bile was hampered by mutating two operon genes, a multidrug resistance (MDR) transporter and a gene of unknown function, suggesting their importance in the bile stress response. The importance of MDR transporters for the bile stress response of *Lb. acidophilus* was also revealed by Pfeiler and Klaenhammer [90]. Indeed, of the ten most highly induced genes in *Lb. acidophilus* in the presence of bile, two were found to encode MDR transporters. These transporters function by extruding structurally unrelated compounds from the cell including antibiotics and bile salts [90]. MDR transporters were also identified in *B. longum* and *B. breve* following exposure to sub-inhibitory concentrations of bile [91]. Expression of the MDR transporter from *B. longum* in *E. coli* conferred bile resistance on the heterologous host when exposed to 3% bile [91]. However, the modified strain exhibited a reduced growth rate; hence the authors suggest that production of the MDR transporter is toxic for the cells. A gene responsible for S-layer production in *Lb. acidophilus* ATCC 4356, *slpA*, was found to be induced in bile concentrations ranging from 0.01-0.05% [92]. S-layer proteins are external bacterial structures which have been associated with protection against hostile environmental elements and the establishment of *Lb. acidophilus* in the GIT [93-95]. However, the expression of *slpA* in 0.1% bile was lower than that recorded in 0.02 and 0.05% bile although the level of S-layer protein on the cell surface increased in concentration. The authors therefore suggest that *slpB*, rather than *slpA*, may be expressed during unfavorable growth conditions [92]. A putative aggregation-promoting factor (Apf) of *Lb. acidophilus* NCFM has been linked to survival of the strain during passage through the GIT and may even be involved in bacterium-host interactions [96]. Indeed, a Δ*apf* deletion mutant was much more susceptible to bile and detergent, and survival rates of the mutant strain were decreased in simulated gastric juice compared to the control. Moreover, adherence of stationary phase mutant cells to an intestinal epithelial cell line was reduced. Overall, the results of the study suggest that Apf-like proteins are important for the gastrointestinal robustness of *Lb. acidophilus*.

## Holistic approaches to understanding and exploiting the probiotic stress response

The general global cellular response of probiotic bacteria to environmental stresses can probably be grouped into six broad biological categories based on differential expression of the associated genes and on the encountered stress(es): (i) stress response genes, (ii) genes involved in energy metabolism, (iii) transcription and translation associated genes, (iv) genes involved in nucleotide metabolism and amino acid biosynthesis genes, (v) cell envelope and cell wall-associated genes, (vi) genes which have no assigned function. For example, whole genome DNA microarrays exploited to study the complete cellular response of *B. longum* NCC2705 to heat treatment of 50^o^C for 3, 7 and 12 minutes revealed that genes involved in cell growth (ribosomal proteins, aminoacyl-tRNA synthetases, genes involved in cell division etc.) were markedly repressed, which as the authors suggest may represent a strategy to conserve energy which can then be directed towards protection mechanisms in the cell [97]. The most highly and rapidly induced genes included *dnaK*, *grpE*, *dnaJ* and the transcriptional repressor HspR. Genes encoding *groEL* and *groES* and the transcriptional regulator HrcA were induced but at lower levels. Numerous transcriptional regulators were induced which included 2 two-component response regulators and the corresponding sensor histidine kinases. Ten LacI-type sugar-responsive-repressors were up-regulated and genes involved in the SOS response were also induced including the transcriptional repressor LexA and genes encoding RecA, RecX and RecN as well as the *trans*-translation machinery. This latter mechanism involves a ribonucleoprotein complex made up of tmRNA (with properties of a tRNA and mRNA-encoded by *ssrA*) and the protein SmpB. Stalled translational complexes serve as the target for tmRNA-SmpB which ‘rescues’ the ribosome and tags the nascent polypeptide and mRNAs for degradation [98]. While *ssrA* was constitutively expressed, the gene encoding SmpB was highly induced following heat-treatment in *B. longum*.

An investigation into the global stress response of *B. breve* UCC2003 to moderate and severe heat treatment as well as osmotic, solvent and oxidative stress revealed that an interactive regulatory network controls the stress response in this strain [99,100]. Interestingly, exposure to moderate temperatures of 42^o^C and 44^o^C for 1 hour resulted in the induction of 5 and 17 genes, respectively and down-regulation of 11 and 92 genes, respectively. In contrast, 267 genes were induced during severe heat treatment (47^o^C) while 266 genes were down-regulated. Under conditions of severe heat shock, genes belonging to carbohydrate transport and metabolism, energy production and conversion and nucleotide transport and metabolism were negatively regulated. Of the up-regulated genes only a fraction were involved in protein misfolding and DNA damage. Based on the overall results, the authors proposed a model interactive regulatory gene network for the bifidobacterial stress response whereby the negative regulator HspR controls the SOS response and the ClgR regulon, which in turn is regulated by and regulates the negative regulator HrcA [99,100].

Previous studies have suggested that different bifidobacteria use NADH peroxidase to prevent the accumulation of H_2_O_2_ (101,102,103), however, none of the sequenced genomes to date contain gene analogs for this enzyme. Interestingly, a recent proteomic study of the oxidative stress-related responses of *B. longum* BBMN68 revealed a change in the expression of 51 protein spots following oxygen exposure revealing a distinctively different set of detoxification proteins compared to other anaerobes (104). Proteins involved in protecting proteins, DNA and RNA were identified including alkyl hydroperoxide reductase (AhpC), pyridine nucleotide-disulfide reductase (PNDR), and DNA oxidative damage-protective proteins including DNA-binding ferritin-like protein (Dps), ribonucleotide reductase (NrdA) and NTP phosphohydrolases (Mut1). The activity of Dps in oxidative stress protection was confirmed by *in vitro* and *in vivo* studies. Indeed, *in vitro* studies revealed that Dps binds DNA to protect it from oxidative degradation and over-expression of the protein in *E. coli* increased survival of the cells under oxidative challenge.

Adapting *Lb. casei* ATCC 334 to a broad range of acid stresses to improve the acid tolerance of the strain demonstrated that exposure to pH 4.5 for 10 or 20 minutes resulted in the highest survival [84]. Assessment of the transcriptional responses of the strain following 5 and 20 minutes of exposure to this pH revealed a dramatic increase in the number of responsive genes following the 20 minute treatment (320 genes with altered expression levels). The majority of genes were down-regulated. This was particularly apparent for genes involved in information storage and processing including translation, ribosomal structure and biogenesis, transcription, DNA replication, recombination and repair as well as genes involved in cellular processes such as protein turnover and remarkably stress response genes and those involved in cell secretion and cell envelope biogenesis. Up-regulated genes included the malolactic enzyme (as discussed earlier) and genes involved in amino acid transport and metabolism, including those involved in the transport of histidine as previously discussed. Interestingly, genes involved in the mobile DNA elements category were also up-regulated following acid adaptation for 20 minutes. Of the poorly characterized up-regulated genes, three were associated with phospholipid turnover: an acetyl transferase, an esterase, and a putative membrane-associated phospholipid phosphatase. Acid-adapted cells had higher total percentages of saturated and cyclopropane fatty acids in the cytoplasmic membrane than control cells which may be linked to the up-regulation of the three phospholipid-associated genes. Other studies have also demonstrated that acid stress induces changes in the cytoplasmic membrane fatty acid content in *Lactobacillus*[105,106]. Moreover, several stresses have been shown to cause an increase in the concentration of cyclopropane fatty acids in the cell membrane including heat stress [107], osmotic stress [108], and bile stress [109,110]. Indeed, using both transcriptomics and strategic proteomics approaches which also enabled a study of cell surface properties (surfome) Koskenniemi et al., [109] demonstrated the up-regulation of a gene encoding cyclopropane-fatty-acyl-phospholipid synthase in *Lb. rhamnosus* GG in response to bile stress, although the level of up-regulation was not deemed to be statistically significant. This study also demonstrated that bile shock resulted in the repression of EPS-encoding genes. The authors postulated that EPS serves to protect *Lb. rhamnosus* GG cells from the harsh environmental conditions of the stomach. However, the presence of bile serves as a signal of gut entrance and hence down-regulation in EPS production to enable better adherence of the bacterium to intestinal cells. Genes involved in the D-alanylation of the negatively charged lipotechoic acids were also up-regulated in response to bile stress which was also observed for *Lb. plantarum*[111]. Such a strategy serves to increase the positive surface charge, and as the authors suggest possibly serves to repulse the cationic compounds in bile. Indeed, alteration of surface charge has also been associated with resistance to cationic peptides [112,113] and we recently observed the same phenomenon in response to bacteriophage challenge in *L. lactis* (unpublished). Several two-component systems, multi-drug transporters, the F_1_F_0_-ATP synthase and a bile salt hydrolase were also up-regulated in response to bile stress as well as several chaperones and proteases directly involved in the stress response.

## Discussion

The ability of probiotic bacteria to survive the harsh environments encountered during processing and gastrointestinal transit has been a major factor in their selection criteria. Indeed, induction of the probiotic stress response through pre-adaptation strategies may not always ensure the improved performance of a strain in compromising environments. For example, exposing *B. longum* DJO10A to sub-lethal stresses of acid, cold and centrifugation before addition to Cheddar cheese as a starter adjunct did not influence the viability of the culture during Cheddar cheese ripening [114]. Moreover, exposure of *Lb. acidophilus* La-5, *Lb. rhamnosus* GG and *Lb. fermentum* ME-3 to temperature, acid or bile stress did not positively affect survival of the strains in a gastrointestinal tract simulator, with a marked decrease in cell numbers for all three strains after the bile phase [115]. In addition, the biological efficacy of probiotic cells may be compromised following exposure to stress. Indeed, spray drying was recently shown to negatively influence the adhesion capacity of *Lactobacillus kefir* 8348 but not *Lb. plantarum* 83114 or *Lb. kefir* 8321 to intestinal cells [116]. Developing a molecular toolbox, whether through patho-biotechnology, targeting indigenous defense strategies of lactic acid bacteria or indeed other bacterial groups, should ensure that the most biologically/functionally active strains can be confidently selected for probiotic development. Indeed, we have already seen how probiotic robustness can be dramatically improved by targeting even a single mechanism. By targeting several cellular defense mechanisms in one strain we should be able to develop designer organisms with the capacity to overcome the plethora of stresses presented during processing and *in vivo* survival.

However, despite the advantages of using such approaches this field of science is not without its limitations. Probiotics which have been enhanced in this way are genetically modified organisms and with the exception of the United States and Canada, there is still uncertainty in the public arena towards the use of genetic manipulation. Moreover, the use of pathogen derived genes in genetically modified probiotics through patho-biotechnology is a concept that undoubtedly consumer and regulatory groups may find hard to accept. Yet despite this, designer probiotics offer huge potential for both technological and clinical applications. For example, Sleator and Hill [117] suggest that ‘bioengineered probiotics’ may provide a safer alternative to attenuated pathogens which are currently used as vaccine delivery platforms or may be used as novel drug delivery vehicles. Moreover, one of the major obstacles associated with genetic manipulation to date is that the benefits of the technology rarely benefit the consumer but rather serve to maximize corporate profit. Genetically modified probiotics, on the other hand, should directly benefit the consumer. However, studies which evaluate the safety of engineered probiotics are crucial if the technology is to gain acceptance. For this reason continued investigation and understanding of the bacterial stress response is a highly worthwhile endeavor in probiotic science providing strategies for scientists to manipulate probiotics to their full potential. In this way next-generation probiotic cultures will be better equipped to face technological and gastrointestinal challenges, (which at the moment can be a rate-limiting step in probiotic selection) as well as meeting medical demands.

## Competing interests

The authors declare that they have no competing interests.
